# RAB26 contributes to the progression of non-small cell lung cancer after being transcriptionally activated by SMAD3

**DOI:** 10.1080/21655979.2022.2051853

**Published:** 2022-03-16

**Authors:** Haixia Ren, Bo Yang, Mingjiang Li, Chunling Lu, Xiaoping Li

**Affiliations:** a Department of Pharmacy, Tianjin First Central Hospital, School of Medicine, Nankai University, Tianjin, China; b Department of Thoracic Surgery, Tianjin First Central Hospital, School of Medicine, Nankai University, Tianjin, China; c Department of Pharmacy, Liaoyang, Liaoning, China

**Keywords:** Non-small cell lung cancer, RAB26, SMAD3

## Abstract

Non-small cell lung cancer (NSCLC) accounts for 85% of all cases of lung cancer, which constitutes the leading cause of cancer mortality. RAB26, a member of Rab GTPase superfamily, has been suggested to play a role in the tumorigenesis of NSCLC. The present work aimed to explore whether and how RAB26 contributed to the progression of NSCLC. NSCLC cell line A549 was transfection with short hairpin RNA (shRNA) or overexpression (Ov) vector to knockdown RAB26 or overexpress SMAD3, respectively. Then the malignant processes of A549 cells including proliferation, migration, invasion and apoptosis were evaluated by CCK-8, colony formation, wound-healing, transwell and TUNEL assays, respectively. Expression of proteins involved in these processes was measured by western blot. A549 xenograft mice model was established to confirm the effect of RAB26 silence on NSCLC progression in vivo. The relationship between RAB26 and SMAD3 was analyzed by bioinformatics and then verified by dual-luciferase reporter and chromatin immunoprecipitation (ChIP) assays. We found that silence of RAB26 inhibited the proliferation, migration and invasion but promoted apoptosis of A549 cells. In vivo studies revealed that the tumor growth of A549 xenograft was markedly suppressed upon RAB26 silence. Moreover, it was confirmed that SMAD3 bound to the promoter of RAB26 and enhance its expression. Finally, we observed that overexpression of SMAD3 significantly blocked the inhibitory effect of RAB26 silence on NSCLC progression. Collectively, RAB26 may contribute to the progression of NSCLC after being transcriptionally activated by SMAD3.

## Graphical Abstract

**Figure:**
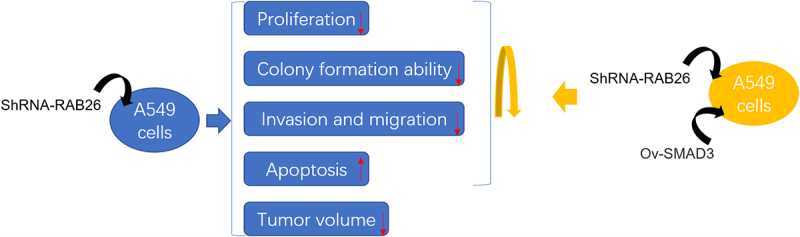

## Introduction

Lung cancer is a cancer with the highest mortality rate worldwide, among which, Non-Small Cell Lung Cancer (NSCLC) accounts for nearly 85% of all cases and is highly malignant [1]. In recent years, although the latest progress has been made in the NSCLC therapies, the 5-year overall survival is still only 11% [2]. And most patients often develop drug resistance during treatment, which is seriously detrimental to the prognosis of patients with NSCLC [3]. Therefore, early detection of tumors is very important for the treatment and prognosis of patients. However, at present, there are still few relevant markers for early screening of NSCLC [4]. Thus, exploring the molecular mechanisms underlying the occurrence and progression of NSCLC and finding novel diagnostic markers are of great significance for the diagnosis of this disease in the early stage and improving the prognosis of patients.

The RAB protein constitutes the largest family of the Ras-like GTPase superfamily and have a key role in membrane vesicles trafficking events [5]. It has been implicated that aberrant expression of RAB GTPases was closely related to tumorigenesis [6]. For instance, upregulated RAB1 has been implicated in multiple cancer types and may predict poor survival [7,8]. RAB27A-mediated exosome secretion was found to aggravate tumor growth, metastasis, and progression [9,10]. In addition, RAB23 overexpression promoted cancer cells invasion and predicted advanced-stage gastric cancer [11].

RAB26 is a member of Rab GTPase superfamily, and is indicated to be up-regulated in NSCLC tissues compared with normal tissue by The Cancer Genome Atlas (TCGA) database. Furthermore, targeted delivery of RAB26 siRNA was suggested to serve as a lung cancer therapy [12]. Importantly, overexpression of RAB26 was found to restore the inhibited tumorigenicity in NSCLC cells [13]. These studies suggested the involvement of RAB26 in NSCLC progression; however, whether and how RAB26 contributed the progression of NSCLC are poorly elucidated.

SMAD3 is an intracellular molecule that can transmit signals from plasma membrane receptors to the nucleus, and especially, a key mediator of classical transforming growth factor-β (TGF-β) signaling pathway, thus playing a crucial role in the TGF-β-mediated transcriptional regulation [14]. SMAD3 expression was up-regulated in lung cancer tissues and cells, and knockdown of it inhibited NSCLC cell migration, invasion and proliferation [15]. A study by Xing et al. suggested that the SMAD3-mediated TGF-β signaling drove the epithelial-mesenchymal transition (EMT) and metastasis of NSCLC [16]. The involvement of SMAD3 in NSCLC progression has also been reported by other studies [17,18].

In this study, we predicted that SMAD3 could bind to the promoter of RAB26 to regulate the malignant processes of NSCLC cells. This study aimed to investigate the effect of RAB26 on the malignant processes of NSCLC cells through the regulation of SMAD3. RAB26 interference plasmid was transfected into the NSCLC cells with or without SMAD3 overexpression plasmid to observe the changes of biological behaviors of NSCLC cells. In addition, RAB26 interference plasmid was also transfected into the mice to observe the changes of tumor growth.

## Materials and methods

### Cell culture and transfection

Human NSCLC cell line A549 (cat. no. CCL-185; ATCC, USA) were cultured in DMEM (Gibco; Thermo Fisher Scientific, Inc.) containing with 10% fetal bovine serum (FBS; Gibco; Thermo Fisher Scientific, Inc.) and 1% antibiotics (Gibco; Thermo Fisher Scientific, Inc.) in a 5% CO_2_ incubator at 37°C.

To knockdown RAB26 expression using short hairpin RNA (shRNA) or overexpress SMAD3 using overexpression (Ov)-SMAD3 in A549 cells, cells were cultured to 70% confluence, then transfected with shRNA against RAB26 (shRNA-RAB26-1 or shRNA-RAB26-2) or Ov-SMAD3 vector that constructed by inserting the human SMAD3 cDNA into pcDNA3.1 cloning vector (ThermoFisher, USA). A scrambled shRNA and empty pcDNA3.1 (GenePharma, Shanghai, China) were used as negative controls (shRNA-NC and Ov-NC). All transfections were performed using lipofectamine 3000 (Invitrogen; Thermo Fisher Scientific, Inc.) referring to the manufacturer’s instruction. At 48 h post-transfection, transfection efficiency was validated by western blot and RT-qPCR assay.

### Cell viability measurement

Cell viability was measured by cell counting kit-8 (CCK-8, cat. no. C0037, Beyotime) assay. Briefly, control or transfected A549 cells were seeded onto 96-well plates at the density of 5 × 10^3^ cells/well and maintained for different hours (24, 48 and 72 h). Thereafter, with the addition of 10 μL of CCK-8 solution to each well, cells were incubated in the dark at 37°C for another 2 h. Finally, the absorbance was detected at 450 nm using a Multiscan MS spectrophotometer (Labsystems) to evaluate cell viability.

### Colony formation assay

For colony formation assay, control or transfected A549 cells were seeded onto 6-well-plates with the density of approximately 300 cells/well and cultured for 2 weeks. Afterward, the colonies in the plates were fixed with 4% of paraformaldehyde and stained using 0.5% crystal violet for 30 min. Finally, colonies were observed and counted.

### Cell migration and invasion assays

Wound-healing assay was employed for detecting cell migration. Cells were seeded onto 6-well plates and cultured to about 80–90% confluence. Afterward, a pipette tip was employed to generate wound by scratching the cell monolayer. Then, cells were incubated in normal DMEM to allow the wound gap healing with cells grow. 48 h later, wound healing area was observed under a light microscope (Olympus; magnification ×100).

Transwell assay was used for assessing cell invasion. Control or transfected A549 cells were resuspended in serum-free DMEM and seeded into the upper compartment of a Transwell chamber, which was pre-coated with matrigel. The lower chamber was filled with DMEM containing 10% FBS. 24 h later, cells that invaded into the lower chamber were fixed with 4% formaldehyde and stained with 0.1% crystal violet. The invaded cells were observed under a light microscope (Olympus; magnification ×100).

### TUNEL staining

Cell apoptosis was observed using Tunel kit (cat. no. C1086, Beyotime). Briefly, control or transfected A549 cells were fixed with 4% paraformaldehyde at room temperature for 30 min, then treated with 1% Triton X-100/10 mM PBS for 5 min. After being incubated with 50 μL of TUNEL detection solution at 37°C for 1 h avoiding the light, cells were exposed to DAPI for stain the nucleus. Finally, observation was performed under a fluorescence microscope (Olympus; magnification ×200).

### Western blot

Total proteins from A549 cells and tumor tissues were isolated using RIPA buffer (Beyotime) on ice. Then equal amount of protein samples were separated by 10–12% SDS-PAGE and transferred to PVDF membranes (Millipore), and then blocked with 5% bovine serum albumin (BSA; Beyotime). After being incubated with primary antibodies at 4°C overnight, samples were subjected to goat anti-rabbit HRP-conjugated secondary antibody (ab6721, 1:10,000, Abcam) at room temperature for 2 h. The bands were finally visualized by an ECL kit (Thermo Scientific), and then intensity was quantified by Image J 1.51 software (National Institutes of Health). Primary antibodies used included: RAB26 (ab198202; 1:000; Abcam), matrix metalloproteinases (MMP)2 (ab92536; 1:1000; Abcam), MMP7 (ab216631; 1:1000; Abcam), Bcl-2 (ab182858; 1:2000; Abcam), Bax (ab32503; 1:5000; Abcam), cleaved caspase 3 (#9661; 1:1000; Cell signaling pathway), caspase 3 (#9662; 1:1000; Cell signaling pathway), Bim (ab32158; 1:1000; Abcam), APAF-1 (ab234436; 1:1000; Abcam), Ki67 (ab16667; 1:1000; Abcam), proliferating cell nuclear antigen (PCNA; ab92552; 1:1000; Abcam), SMAD3 (ab208182; 1:1000; Abcam) and GAPDH (ab181602; 1:5000; Abcam).

### RT-qPCR

Total RNA from A549 cells was obtained with TRIZOL reagent (Invitrogen). With the application of PrimeScript™ RT Master Mix kit (Takara) and SYBR Green kit (Takara), cDNA was synthesized, and then Real-time quantitative PCR (qPCR) was performed. GAPDH was used as an internal reference and the calculation of the relative gene expression was performed using the 2^−ΔΔCq^ method [19]. The thermocycling conditions were as follows: Initial denaturation at 95°C for 180 sec, followed by 40 cycles at 95°C for 10 sec, 60°C for 20 sec and 72°C for 30 sec. Primers sequences are listed as follows:

RAB26, F:5’-GTCTGCTGGTGCGATTCAAG-3’, R:5’-GCATGGGTAACACTGCGGA-3’;

SMAD3, F:5’-CGCACTGACCATAAGAGCAA-3’, R: 5’-CCATCCAGGGACTCAAACG-3’;

GAPDH, F:5’-AATGGGCAGCCGTTAGGAAA-3’, R:5’-GCGCCCAATACGACCAAATC-3’.

### Xenograft tumor

The Ethics Committee of Tianjin First Central Hospital has approved the animal experiments in this study. A total of 15 6–8 weeks-old BALB/c nude mice were purchased from the Beijing Vital River Laboratory Animal Technology. To establish NSCLC xenograft model, mice were randomly assigned into 3 groups (n = 5 per group), and then were subcutaneously injected with A549 cells (A549 group), A549 cells that transfection with shRNA-NC (shRNA-NC group) or A549 cells that transfection with shRNA-RAB26 (shRNA-RAB26 group) at the density of 5 × 10^6^ cells in 100 mL serum-free medium. The mice body weight and volume of tumors were recorded every 3 days, and at the end of the experiment (21 days later), the mice were sacrificed by cervical dislocation under anesthesia and the tumors were weighted and collected for further western blot analysis.

### Dual-luciferase reporter assay

To investigate the interaction between SMAD3 and RAB26, the RAB26 full-length promoter (FL), E1 deleted promoter (E1 Del) or E2 deleted promoter (E2 Del) was cloned into the PGL3-luc luciferase reporter vector (Promega), then co-transfected with Ov-SMAD3 or control vector (empty pcDNA3.1) into A549 cells using lipofectamine 3000 (Invitrogen). Luciferase activities were detected at 48 h post-transfection via the Dual-luciferase Reporter Assay System (Promega). The reporter activity was represented by the ratio of Firefly luciferase activity/Renilla luciferase activity, then normalized to control group.

### Chromatin immunoprecipitation (ChIP) assay

The SimpleChIP Enzymatic Chromatin IP Kit (#9002; Cell Signaling) was utilized to perform ChIP in accordance with the manufacturer’s instruction. In brief, A549 cells were cross-linked by 1% formaldehyde on ice for 0.5 h. After being washed with PBS, cells were sonicated on ice to breakdown the chromatin into an average length of 1000 bp. IP was carried out with the antibody against SMAD3 (ab208182), IgG (ab172730) was used as a negative control (Abcam, USA). Afterward, the complex was collected by protein A agarose beads, and the DNA was eluted from the immunoprecipitated complexes on the agarose beads. Precipitated DNAs were identified by quantitative PCR.

### Bioinformatics tools

The potential-binding sites between SMAD3 and RAB26 promoter was predicted by transcription factor (TF) database (http://bioinfo.life.hust.edu.cn/).

### Statistical analysis

All experiments were performed in triplicate. The Student’s t-test was employed to analyze the difference between two groups with p value less than 0.05 considered significance. The differences among the multiple groups were accomplished by one-way ANOVA followed by Tukey’s Test. Data are presented as the mean ± standard deviation.

## Results

### RAB26 silence suppresses proliferation, migration, invasion, and promotes apoptosis of A549 NSCLC cells

**Figure 1.**
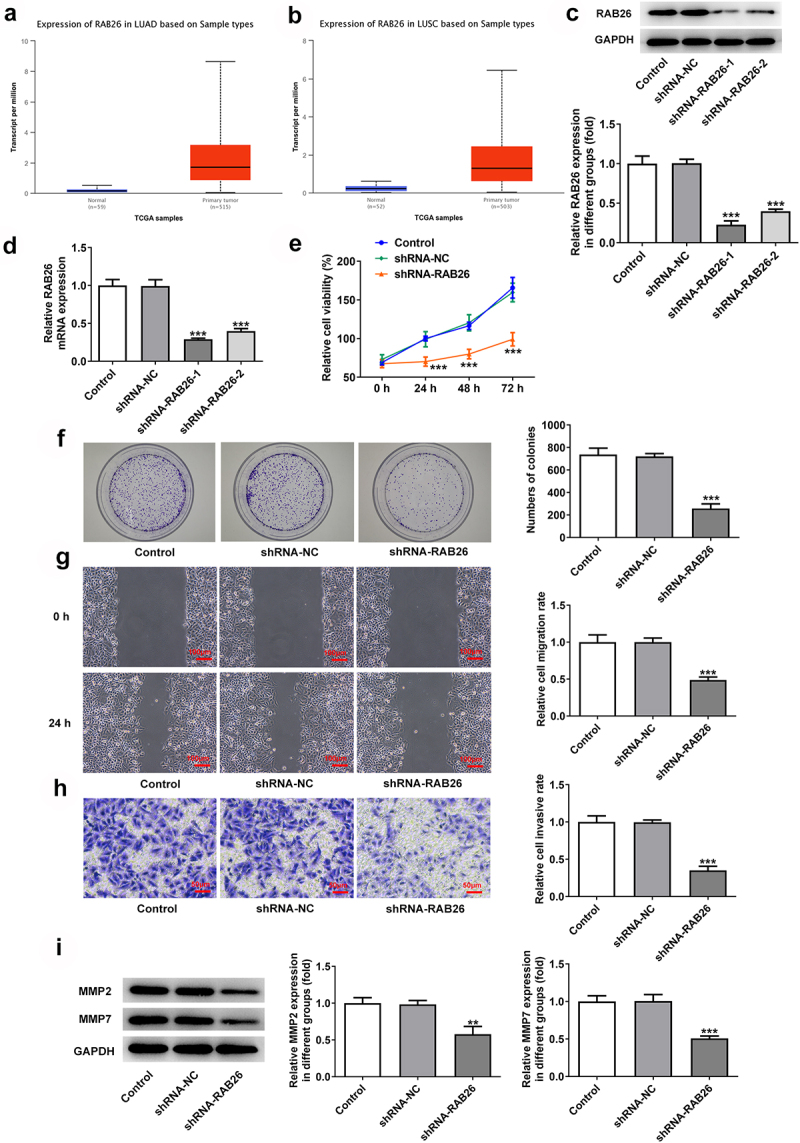
RAB26 silence inhibits A549 cells proliferation, migration and invasion.

**Figure 2.**
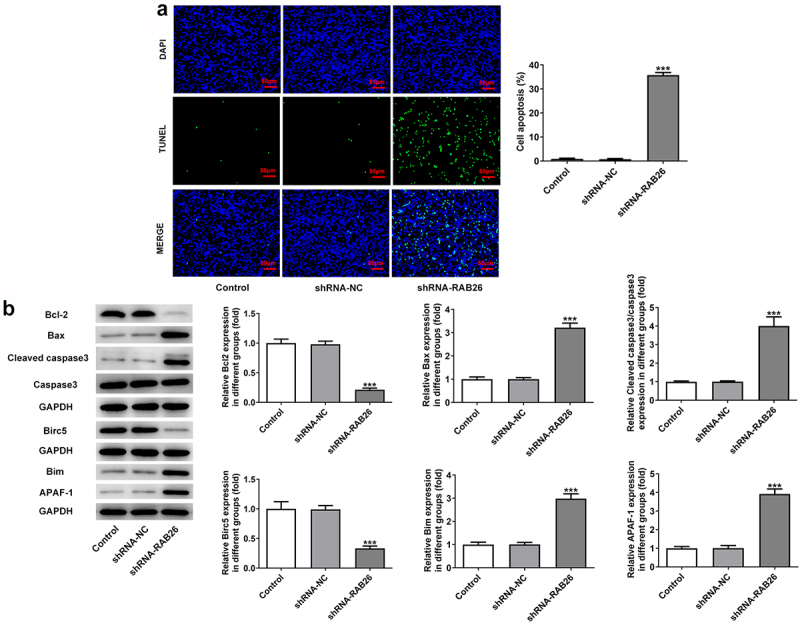
RAB26 silence promotes A549 cells apoptosis.

Firstly, to evaluate the role of RAB26 in NSCLC progression, RAB26 expression was knockdown in A549 cells via transfection of shRNAs. The expression of RAB26 was increased in tumor tissues of NSCLC including LUAD and LUSC (Figure 1a,b). The knockdown efficiency was determined by RT-qPCR and western blot assays, and results showed that shRNA-RAB26-1 exhibited better transfection efficiency; therefore, it was chosen for subsequent experiments (Figure 1c,d). By detecting the cell viability, it was found that A549 cells that transfection with shRNA-RAB26 showed significant lower cell viability than cells that transfection with shRNA-NC (Figure 1e). Similarly, the number of colonies in shRNA-RAB26 group was obviously smaller than that in shRNA-NC group (figure 1f). RAB26 silence also caused markedly reduced migration distance in wound-healing assay and decreased invaded cells in transwell assay, when compared with shRNA-NC (Figure 1g,h). Consistently, the expression of MMP2 and MMP7 was also down-regulated by RAB26 silence (Figure 1i). TUNEL staining was then used to evaluate cell apoptosis, illustrating that shRNA-RAB26 resulted in greatly increase in cell apoptosis (Figure 2a). RAB26 silence also led to decreased expression of Bcl-2 and Birc5, but increased expression of Bax, cleaved-caspase3/caspase3, Bim and APAF-1 (Figure 2b).

A, the expression of RAB26 in LUAD tumor tissues. B, the expression of RAB26 in LUSC tumor tissues. C, the protein expression of RAB26 in A549 cells before and after transfection with indicated vectors was detected by western blot assay. D, the mRNA level of RAB26 in A549 cells before and after transfection with indicated vectors was detected by RT-qPCR. E, cell viability of control or transfected A549 cells at 24, 48 and 72 h post transfection was measured by CCK-8. F, representative images for colony formation assay of A549 cells. G, wound-healing assay was employed to assess cell migration. H, cell invasion was detected by transwell assay. I, the protein expression of MMP2 and MMP7 was measured by western blot. **P < 0.01 and ***P < 0.001 vs shRNA-NC.

A, control or transfected A549 cell apoptosis was measured by TUNEL staining (scale bar = 50 μm). B, the protein expression of Bcl-2, Bax, cleaved-caspase3/caspase3, Birc5, Bim and APAF-1 was evaluated by western blot assay. ***P < 0.001 vs shRNA-NC.

### RAB26 silence inhibits tumor growth of A549 NSCLC xenograft

**Figure 3.**
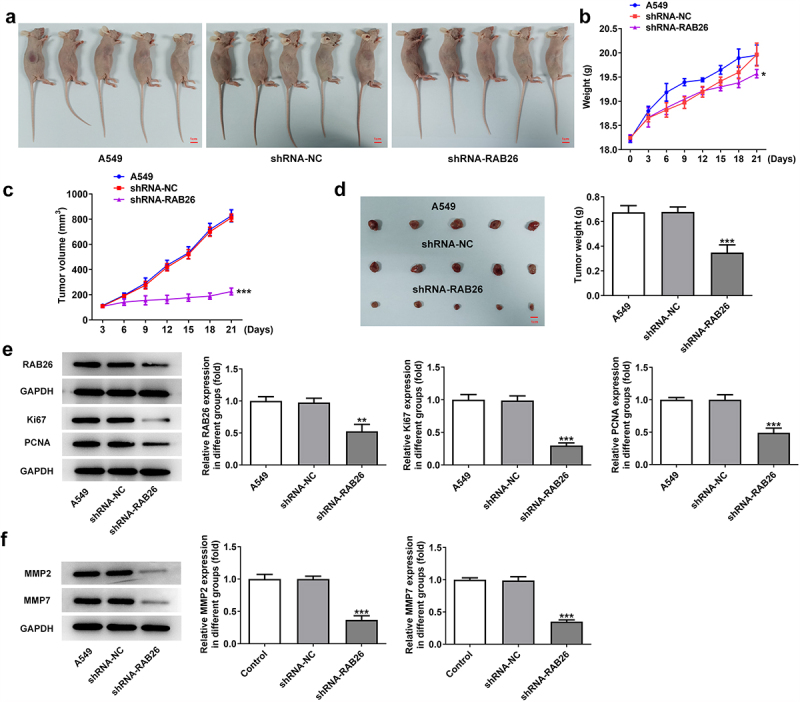
RAB26 silence suppresses the growth of A549 xenograft tumor in vivo.

**Figure 4.**
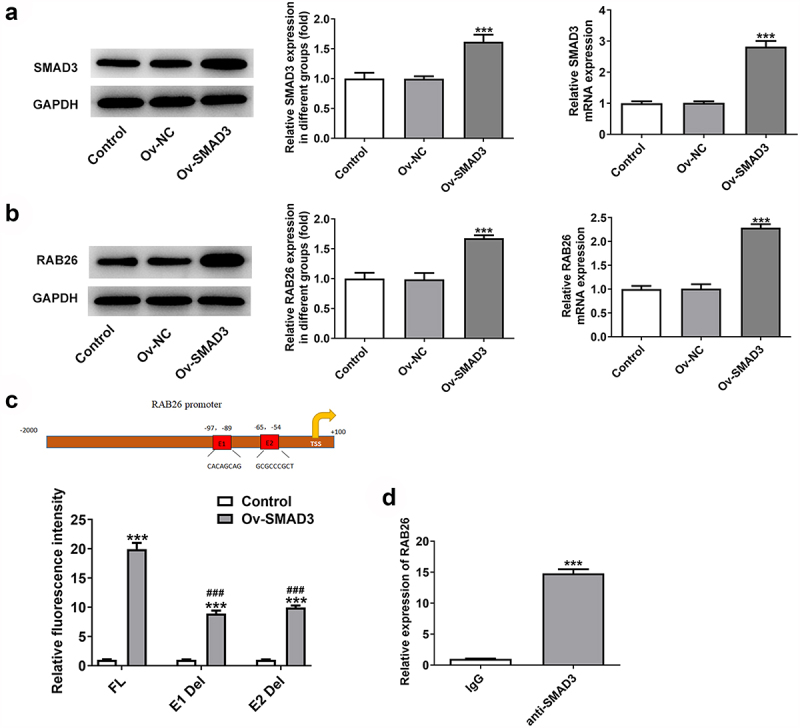
SMAD3 binds to RAB26 promoter to activate its expression.

Next, to validate the anti-cancer effect of RAB26 silence on NSCLC in vivo, A549 NSCLC cells xenograft animal model was established (Figure 3a). The body weight and tumor volumes of mice in each group was monitored, and results showed that RAB26-silenced A549 xenograft tumor exhibited significantly reduced tumor volume growth than that in shRNA-NC group (Figure 3b-c). Besides, the tumor weight was also considerably decreased in shRNA-RAB26 group (Figure 3d). The protein expression of RAB26, Ki67, PCNA as well as MMP2 and MMP7 in xenograft tumor tissues from each group was also determined by western blot assay. As shown in Figure 4e,4f, shRNA-RAB26-transfected A549 xenograft tumors exhibited significantly decreased expression of the above proteins as compared with shRNA-NC-transfected A549 xenograft tumors (Figure 3e,f).

A, A549 cells or cells that transfected with shRNA-NC or shRNA-RAB26 were injected into mice to establish NSCLC xenograft tumor model. B-C, mice body weight and tumor volume were recorded every three days. D, at 21 day post injection, tumors in each group were removed and weighed. E-F, the protein expression of RAB26, Ki67, PCNA as well as MMP2 and MMP7 in xenograft tumor tissues from each group was determined by western blot assay. *P < 0.05, **P < 0.01 and ***P < 0.001 vs shRNA-NC.

A-B, the protein and mRNA expression levels of SMAD3 (A) and RAB26 (B) in control or transfected A549 cells were detected by western blot and RT-qPCR assays. ***P < 0.001 vs Ov-NC. C, dual-luciferase reporter assay was utilized to determine the interaction between RAB26 promoter and SMAD3. ***P < 0.001 vs Control; ^###^P < 0.001 vs FL + Ov-SMAD3. D, ChIP assay was performed to detect the binding between RAB26 and SMAD3, the mRNA level of RAB26 in immunoprecipitated products were identified by RT-qPCR. ***P < 0.001 vs IgG.

### SMAD3 binds to RAB26 to enhance RAB26 expression

Subsequently, the interaction between RAB26 and SMAD3 was verified. As demonstrated in Figure 4a,b, overexpression of SMAD3 using Ov-SMAD3 vector not only effectively up-regulated the expression level of SMAD3 but also enhanced RAB26 protein and mRNA expression levels in A549 cells, suggesting that SMAD3 could activate RAB26 expression. Afterward, the interaction between SMAD3 and RAB26 promoter was validated by dual-luciferase reporter and ChIP assays (Figure 4c,d). Therefore, SMAD3 could bind to RAB26 promoter to activate RAB26 expression.

### SMAD3 overexpression reverses the inhibitory effect of RAB26 silence on NSCLC progression

**Figure 5.**
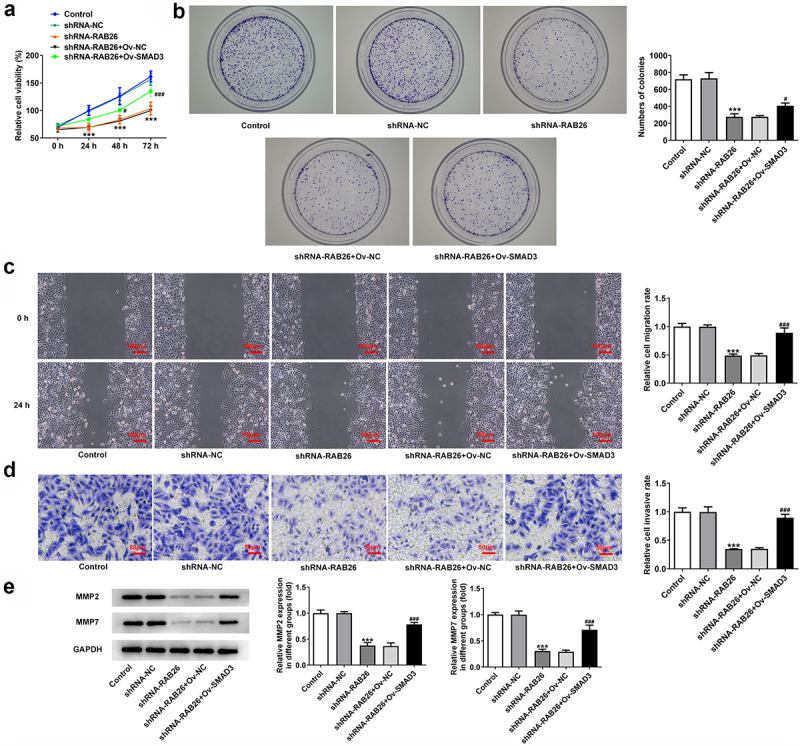
SMAD3 overexpression reverses the inhibitory effect RAB26 silence on A549 cells proliferation, migration and invasion.

**Figure 6.**
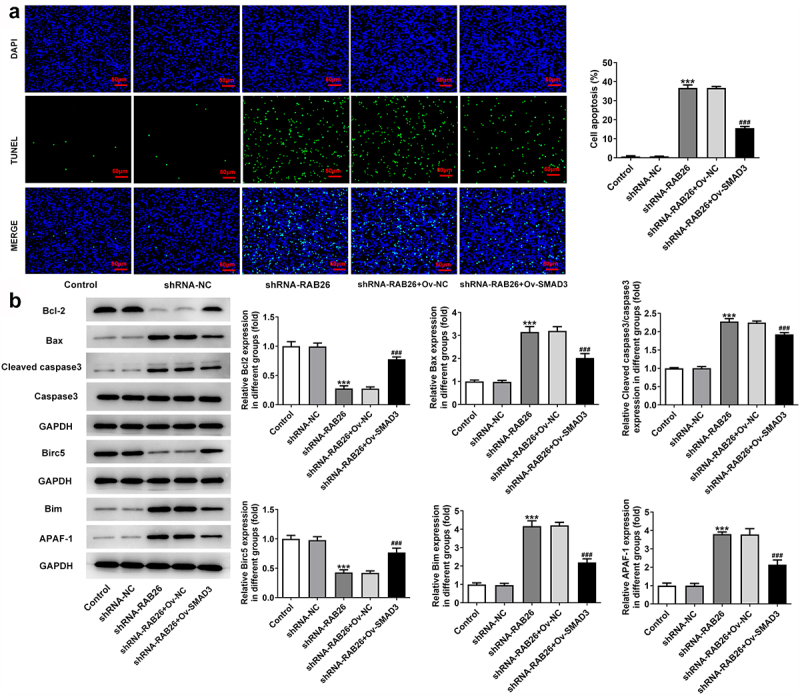
SMAD3 overexpression reverses the promoted effect RAB26 silence on A549 cells apoptosis.

Finally, to explore whether SMAD3-meditaed transcriptional activation of RAB26 was required for the actions of RAB26 on NSCLC, SMAD3 was overexpressed in A549 cells that silenced with RAB26 via transfection with Ov-SMAD3. Results revealed that, compared with Ov-NC, OV-SMAD3 significantly increased the cell viability of shRNA-RAB26-transfected A549 cells (Figure 5a). Also, additional overexpression of SMAD3 in RAB26-silenced A549 cells markedly increased the ability of colony formation (Figure 5b). These data implicated that SMAD3 overexpression reversed the inhibitory effect of RAB26 silence on A549 cells proliferation. In addition, cell migration and invasion were also assessed. Results showed that the decreased migration and invasion capacities of A549 cells caused by RAB26 knockdown were considerably recovered upon SMAD3 overexpression (Figure 5c,d). Consistently, SMAD3 overexpression also restored the expression of MMP2 and MMP7 in A549 cells, which was decreased by RAB26 silence (Figure 5e). Furthermore, results from TUNEL staining illustrated that the increase in cell apoptosis caused by RAB26 silence was significantly blocked after SMAD3 overexpression (Figure 6a). At the same time, SMAD3 overexpression significantly reversed the expression of proteins involved in apoptosis including Bcl-2, Bax, cleaved-caspase3/caspase3, Birc5, Bim and APAF-1, which changed by shRNA-RAB26 (Figure 6b).

A, cell viability of control or transfected A549 cells at 24, 48 and 72 h post transfection was measured by CCK-8. B, representative images for colony formation assay of A549 cells. C, wound-healing assay was employed to assess cell migration. D, cell invasion was detected by transwell assay. E, the protein expression of MMP2 and MMP7 was measured by western blot. ***P < 0.001 vs shRNA-NC;^###^P < 0.001 vs shRNA-RAB26 + Ov-NC.

A, control or transfected A549 cell apoptosis was measured by TUNEL staining (scale bar = 50 μm). B, the protein expression of Bcl-2, Bax, cleaved-caspase3/caspase3, Birc5, Bim and APAF-1 was evaluated by western blot assay. ***P < 0.001 vs shRNA-NC;^###^P < 0.001 vs shRNA-RAB26 + Ov-NC.

## Discussion

In the current study, we observed that RAB26 knockdown effectively inhibited NSCLC progression via suppressing NSCLC cells proliferation, migration, invasion and promoting apoptosis. We also identified SMAD3 as an important regulator that was involved in RAB26-mediated NSCLC progression through binding to RAB26 promoter to activate its expression.

The RAB GTPases are known to orchestrate vesicle-mediated protein transport and RAB26 is a recently identified small GTPase, was originally found to regulate amylase secretion from parotid acinar cells [20]. Since then, RAB26 was gradually reported to participate in lysosomal trafficking and autophagy during the modulation of exocrine granule maturation. For example, RAB26 was implicated to modulate the cell surface transport of α2-adrenergic receptors from the Golgi [21]. RAB26 also coordinated lysosome traffic, mitochondrial localization and exocrine granule maturation [22,23]. A study by Beyenech et al. first showed that RAB26 selectively directed synaptic vesicles into autophagosome structures, indicating the involvement of RAB26 in autophagy pathway [24]. The role of RAB26 in cancer was recently proposed. Qian et al. regarded RAB26 as a potential therapeutic for cancers considering that RAB26 regulated endothelial cell apoptosis and hyper-permeability [12]. Afterward, the role of RAB26 in NSCLC was uncovered by Nianli et al., showing that RAB26 served as a target as SNRPB, which promoted the tumorigenic potential of NSCLC [13]. The data from TCGA reveals the up-regulated expression of RAB26 in NSCLC tissue compared with normal tissue. Nonetheless, the specific role of RAB26 in NSCLC remains elusive. Herein, we found that RAB26 silence significantly inhibited the proliferation, migration, and invasion of NSCLC cells. Further in vivo studies confirmed the inhibitory effect of RAB26 silence on NSCLC tumor growth. Ki67 and PCNA are markers of cancer cells proliferation, high expression of them indicates the tendency to relapse and metastasis. MMPs play an important role in cancer progression and metastasis [25]. It is known that metastasis accounts for the dominant reason of NSCLC-related death. Our results showed that RAB26 silence reduced the expression of Ki67, PCNA, MMP2 and MMP7 in xenograft tumor samples, suggesting the suppression of RAB26 silence on NSCLC growth and metastasis in vivo.

More importantly, we identified SMAD3 as an up-stream target of RAB26. We found that SMAD3 could bind to RAB26 promoter and positively regulate RAB26 expression. To validate whether SMAD3 down-regulation was required for RAB26 silence-mediated anti-cancer effect on NSCLC, we additionally overexpressed SMAD3 in RAB26-silenced A549 cells to observe the changes in malignant processes. In accordance with our speculation, the anti-cancer effect of RAB26 silence on NSCLC cells was markedly abolished by SMAD3 overexpression. Also, there are many studies related to the role of SMAD3 in various cancers. Macrophage-specific silencing of SMAD3 effectively blocks macrophage–myofibroblast transition (MMT), thereby inhibiting cancer-associated fibroblasts (CAF)-mediated cancer progression [26]. The prostate cancer progression was activated when the SMAD3 was upregulated [27]. SMAD3 could activate the TGF-β-induced EMT process of bladder cancer cells [28]. We can also observe that SMAD3 can promote the cancer progression, as demonstrated in this study.

In addition, SMAD3 is a key mediator of canonical TGF-β signaling, which is known to influence tumor progression via regulating cancer cells infiltration, adhesion, EMT and extracellular matrix degradation. In NSCLC, TGF-β signaling is frequently enhanced and promotes EMT and tumor metastasis, whereas SMAD3 inhibition diminished TGF-β-induced EMT in cancer cells [29]. In addition, SMAD3 overexpression was demonstrated to accelerate TGF-β-mediated NSCLC metastasis [30]. In this work, the oncogenic role of SMAD3 in NSCLC via activating RAB26 expression was uncovered. As a result, our study not only uncovered the oncogenic role of RAB26 in NSCLC, but also identified a novel target for SMAD3-mediated cancer progression. Whether SMAD3 could target RAB26 in other cancer cells to regulate cancer progression is of worth exploring.

## Conclusion

Overall, this study for the first time demonstrated that RAB26 silence inhibited NSCLC progression both in vitro and in vivo. Besides, SMAD3-mediated RAB26 transcriptional activation was indispensable for the oncogenic role of RAB26 in NSCLC. These findings unveiled SMAD3/RAB26 axis as a therapeutic target for treating NSCLC.
